# Activation and maturation of peripheral blood T cells in HIV-1-infected and HIV-1-uninfected adults in Burkina Faso: a cross-sectional study

**DOI:** 10.1186/1758-2652-14-57

**Published:** 2011-12-17

**Authors:** Fabrice Tiba, Frans Nauwelaers, Lassana Sangaré, Boubacar Coulibaly, Hans-Georg Kräusslich, Thomas Böhler

**Affiliations:** 1 Department of Infectious Diseases, Virology, University of Heidelberg, Heidelberg, Germany; 2 BD Biosciences, Erembodegem, Belgium; 3 Centre Hospitalier Universitaire Yalgado Ouedraogo, Ouagadougou, Burkina Faso; 4 Centre de Recherche en Santé de Nouna, Nouna, Burkina Faso

## Abstract

**Background:**

We wanted to explore to what extent environmental exposure to immune stimulants, which is expected to be more present in rural than in urban settings, influences T cell activation and maturation in healthy and in HIV-1-infected individuals in Burkina Faso in west Africa.

**Methods:**

The proportion of circulating naïve T cells and the expression of the T cell activation markers, CD95 and CD38, were analyzed by immunophenotyping and three-colour flow cytometry in 63 healthy individuals and 137 treatment-naïve HIV-1-infected subjects from Ouagadougou (urban setting) and 26 healthy adults and 61 treatment-naïve patients from Nouna (rural).

**Results:**

A slightly higher activation level of CD4^+ ^and CD8^+ ^peripheral blood T cells was seen in healthy adults living in Nouna than in those living in Ouagadougou. The percentages of naïve CD45RA^bright ^CCR7^+ ^T cells were not significantly different between both study sites. Taking into consideration that relatively more HIV-1-infected patients in Nouna were in an advanced disease stage, no relevant differences were seen in T cell activation and maturation between patients at both study sites. As expected, the percentage of CD95^+ ^CD4^+ ^and CD38^+ ^CD8^+ ^T cells and the respective antigen density on these cells was significantly higher in patients than in controls in both settings. The percentage of naïve CD8^+ ^T cells was lower in HIV-1-infected subjects than in healthy controls irrespective of the study site, while a lower proportion of naïve CD4^+ ^T cells in patients compared with controls was seen only in Nouna.

**Conclusions:**

Environmentally triggered immune activation may contribute to the increased expression of the activation markers CD95 and CD38 on peripheral blood T cells from healthy adults living in rural versus urban settings in Burkina Faso. T cell activation is further increased in HIV-1-infected individuals due to T cell loss and high plasma viral load levels. The observed variations in T cell activation levels or the proportion of naïve T cells in our study patients, however, are not explained by differences in CD4^+ ^T cell counts or HIV-1 plasma viral load levels alone.

## Background

HIV pathogenesis is characterized by a progressive depletion of CD4^+ ^T cells in the course of the disease and a chronic systemic immune activation associated with a redistribution of T cell maturation phenotypes [1,2]. A similar activation status has been described in healthy individuals living in a tropical environment, albeit less pronounced compared with HIV-infected individuals [3-5]. Environmental stimuli, and in particular frequent intermittent infections, may be the driving force for such activation. They may thus play an important role in the HIV epidemic in Africa by accelerating CD4^+ ^T cell depletion, resulting in faster disease progression compared with the situation in developed countries [6-9].

Previously, we had observed significant differences in the distribution of T cell maturation phenotypes between healthy adults in Burkina Faso [10] and published data from populations in Europe [11]. In the few studies that have addressed the immunological consequences of HIV-1-infection in Africans, the focus has been directed towards the expression of CD38 on CD8^+ ^T cells [12-14]. Upregulation of CD95 in HIV-infected subjects has been associated with disease progression in Europe [15-17], but has not yet been extensively studied in African patients. To increase our understanding of HIV-induced immune alteration in tropical settings, we concomitantly assessed the presence of naïve T cells and the activation level of T cells by expression of both CD95 and CD38 in HIV-1-infected adults, as well as in healthy controls, living in a rural or an urban setting in Burkina Faso. We wanted to explore to what extent environmental exposure to immune stimulants, which is expected to be more present in rural settings, influences T cell activation and maturation in healthy and in HIV-1-infected individuals.

## Methods

Both the National Ethics Committee in Burkina Faso and the Institutional Ethics Committee of the University of Heidelberg approved this study. From May 2008 to September 2009, 137 treatment-naïve HIV-1-infected subjects without previous exposure to single or combined antiretroviral drugs or highly active antiretroviral combination therapy (HAART) who were visiting the outpatient clinic at the Centre Hôpitalier Universitaire Yalgado Ouedraogo (CHUYO) in Ouagadougou, the capital of Burkina Faso, were included (urban setting). In Nouna, 61 treatment-naïve adult patients were recruited at the Centre de la Recherche en Santé de Nouna (CRSN) from January 2009 to September 2009 (rural setting).

All subjects signed an informed consent form prior to entering the study. Fresh blood samples, clinical and socio-demographic data were collected from the patients at regular clinical visits. For comparison of T cell maturation and activation markers between HIV-1-infected patients and control subjects living in urban and rural Burkina Faso, healthy adults were recruited from clinical and laboratory personnel at the CHUYO in Ouagadougou (urban, n = 63) and at the CRSN in Nouna (rural, n = 26).

At both study sites, patients and controls were always tested simultaneously during six periods of six to eight weeks each at approximately three-month intervals. In order to minimize preanalytic confounders, venipuncture, blood staining and fluorescence-activated cell sorting (FACS) analyses were done in the same laboratory in the respective study sites. Storage time never exceeded four hours. Fifty microliters of peripheral blood anticoagulated with K_3_-EDTA were used to determine absolute numbers of CD4^+ ^and CD8^+ ^T cells in the FACSCount system (BD Biosciences, San Jose, CA, USA) according to the manufacturer's instructions. Plasma obtained from 125 patients in Ouagadougou and 61 patients in Nouna was used for the quantification of viral particles with the Abbott m2000rt RealTime HIV-1 test (Abbott, Chicago, IL, USA) at the Laboratory of Bacteriology and Virology of CHUYO after short centrifugation of 5ml whole blood at 3000rpm. The detection limit was 50 HIV-1 RNA copies per ml of plasma.

Multiparametric flow cytometry was performed using a three-colour FACSCalibur flow cytometer (FCM) in Ouagadougou and a FACScan FCM in Nouna (both from BD Biosciences). Measurements were done with cocktails of fluorochrome-labelled monoclonal antibodies (all from BD Biosciences) aliquoted in 3ml BD FACS tubes and 100 μl venous whole blood anticoagulated with K_3_-EDTA exactly as specified by the manufacturer. CaliBrite beads (BD Biosciences) were used to set the instrument on a regular basis. Instrument settings were controlled longitudinally and remained constant during the study. Data acquisition for each analysis was stopped manually when at least 10,000 events were counted in the predefined gate. List mode data from all samples were analyzed using the CellQuest Pro software, version 5.2 (BD Biosciences).

**Figure 1:**
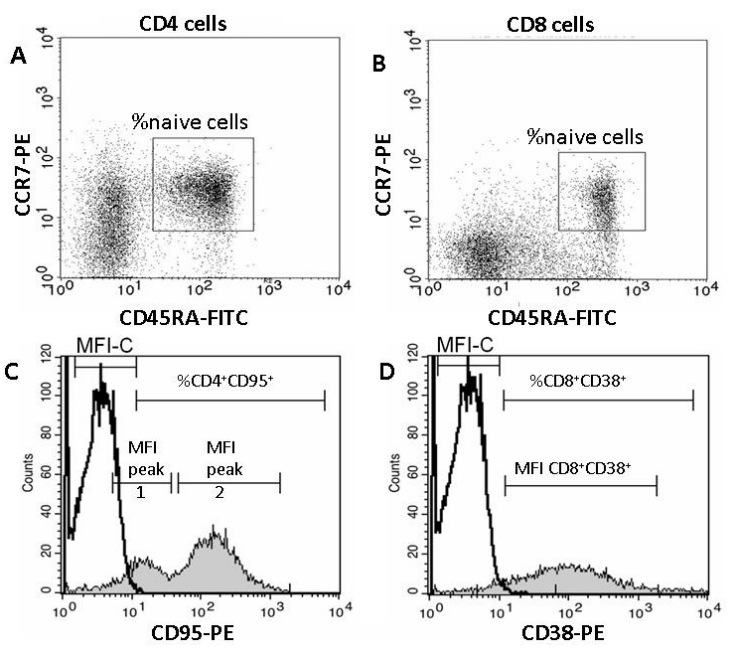
**Flow-cytometric assessment of the percentage of naïve T cells and the expression of T cell activation markers**. For the determination of naïve T cell frequencies, whole blood was stained with anti-CD4 or -CD8 together with anti-CCR7 and anti-CD45RA antibodies. Panels A and B show dot plots of CD4^+ ^and CD8^+ ^T cells with their respective gating of the frequencies of naïve cells as shown in the region markers. For the determination of activated T cell frequencies, whole blood was stained with anti-CD3 and anti-CD4 or anti-CD8 together with anti-CD95 or anti-CD38 antibodies, respectively. Panels C and D show histogram plots of CD4^+ ^and CD8^+ ^T cells expressing CD95 and CD38 activation markers, respectively. The peak of unstained control is shown in the first decade of each plot. Peaks of CD95^dim ^and CD95^bright ^on CD4^+ ^cell populations were separately analyzed. The expression of CD38 on CD8^+ ^T cells was characterized by a unique peak.

CD4^+ ^and CD8^+ ^naïve and memory T cell subsets were assessed with anti-CD4 or anti-CD8-PerCP, anti-CD45RA-FITC and anti-CCR7-PE conjugated monoclonal antibodies as described [10]. Naïve T cells were identified as CD45RA^bright ^CCR7^+ ^CD4^+ ^or CD8^bright ^lymphocytes [18-20]. T cell activation was determined with anti-CD95-PE, anti-CD3-PerCP and anti-CD4-FITC to assess the expression of CD95 on CD4^+ ^T cells, and anti-CD38-PE, anti-CD3-PerCP and anti-CD8-FITC to assess the expression of CD38 on CD8^+ ^T cells. Figure 1A to 1D shows typical examples of flow-cytometric assessment of the percentage of naïve and activated T cell subpopulations.

The median fluorescence intensity (MFI) of CD95 on CD4^+ ^T cells is characterized by a bimodal distribution with two peaks, i.e., CD95^dim ^and CD95^bright ^[15,21,22] which were analyzed separately. We had shown in the past by magnetic bead isolation and three-colour flow cytometry [10] and by four-colour flow cytometry [21] that the cell populations constituting the CD95^dim ^and the CD95^bright ^peak correspond to resting naïve and activated memory cells, respectively. CD4^+ ^T cells classified as "naïve", "resting", or "not activated" by surface marker analysis using, for example, CD45RA, CD45R0, CD62L or CCR7, may thus express various levels of the activation marker CD95. The expression of CD38 on CD8^+ ^T cells is characterized by a skewed unimodal histogram curve [23,24]; the cursor for MFI determination was set on CD38^+ ^cells. The QuantiBrite test kit (BD Biosciences) was used in all experiments to convert MFI values into antibody-binding capacity (ABC) per cell according to the instructions of the manufacturer. Since calibration curves were generated on each instrument at each site separately using the same batch of QuantiBrite beads, the resulting numerical ABC values were not influenced by the different FCM settings at the different study sites.

Mean values and standard deviations, as well as median values and percentiles, were calculated for each variable using the GraphPad Prism version 5.00 (Graph Pad Inc., San Diego, California, USA). Differences between healthy controls and HIV-1-infected subjects, as well as differences between the different study sites, were analyzed using the non-parametric Mann-Whitney U-test, p values < 0.01 indicated statistical significance. Correlations between T cell activation levels and CD4^+ ^T cell counts or HIV-1 plasma viral load were explored separately in the patient cohorts from Nouna and Ouagadougou by linear regression analysis using Pearson's correlation coefficients (r) and the coefficients of determination (R^2^).

## Results

### Differences between healthy controls living in rural and urban settings

Table 1 shows detailed information on the number, age and gender distribution of HIV-1-infected patients and healthy controls at both study sites. The number of patients with complete multiparameter flow-cytometric measurements varied slightly for each test because the necessary monoclonal antibodies were not always available due to logistic delivery problems in Burkina Faso (exact numbers are given in additional file 1). Healthy adults living in the rural setting showed a higher percentage of CD95^+ ^CD4^+ ^T cells in peripheral blood than the controls in the urban setting (median [10^th ^and 90^th ^percentile]: 91 [77-98] % in Nouna vs. 82 [68-95]% in Ouagadougou, p < 0.01; see also additional file 1).

**Table 1:** Comparison of clinical and immunological parameters between HIV-1-infected and healthy adults living in Burkina Faso

	Nouna (rural)		*p*	Ouagadougou (urban)		*p*
	**Patients**	**Controls**		**Patients**	**Controls**	

n =	61	26	-	137	63	-
Female [n = ; (%)]	44 (72%)	16 (61%)	<0.0001	98 (72%)^c^	25 (40%)*	<0.0001
Age [years; median, range]	35 (30-43)	27 (19-35)	<0.0001	35 (23-48)	28 (20-35)	<0.0001
HIV-1 plasma viral load (log_10 _copies ml^-1^]	5.8 (4.6-6.6) [n = 61]	n.d.	-	5.4 (4.0-6.2)^b^ [n = 125]	n.d.	-
CD4^+ ^T cell count [μl^-1^]	174 (33-314)	n.d.	-	256 (118-387)^b^	n.d.	-
CDC-stage A [n = ; (%)]	01 (02%)	n.d.	-	22 (16%)^c^	n.d.	-
CDC-stage B [n = ; (%)]	23 (38%)	n.d.	-	55 (40%)^c^	n.d.	-
CDC-stage C [n = ; (%)]	37 (60%)	n.d.	-	60 (44%)^c^	n.d.	-

All patients were "treatment-naïve", i.e., without known or reported previous exposure to single or combined antiretroviral drugs or highly active antiretroviral combination therapy (HAART). If not otherwise indicated, median values are shown with 10^th ^and 90^th ^percentiles in parentheses. The Mann-Whitney U-test was used to compare patients and controls in the rural and urban setting, as well as patients in the rural and urban setting and controls in the rural and the urban setting separately.

Abbreviations: p - p value for the difference between patients and controls; CDC - Centers of Disease Control and Prevention immunologic staging; n.d. - not done

^a^p <0.05, ^b^p <0.001, ^c^p <0.001 indicating statistically significant differences between HIV-1 infected subjects in Ouagadougou (urban) and HIV-1 infected subjects from Nouna (rural)

*p < 0.05; **p < 0.01; ***p < 0.001, indicating statistically significant differences between healthy controls in Ouagadougou (urban) and healthy controls in Nouna (rural)

The percentage of CD38^+ ^CD8^+ ^T cells was higher in Nouna (83 [63-98]%) than in Ouagadougou (63 [36-83]%; p < 0.0001) and also the expression level (ABC) of CD95 on CD95^dim ^CD4^+ ^T cells and of CD38 on CD38^+ ^CD8^+ ^T cells was significantly higher in Nouna (additional file 1). The percentages of naïve CD45RA^bright ^CCR7^+ ^CD4^+ ^and CD8^+ ^T cells were not significantly different between both study sites. However, there was a moderate but significant correlation between the percentage of CD95^+ ^CD4^+ ^T cells and the percentage of naïve CD4^+ ^T cells in healthy adults from Ouagadougou and a tendency towards a similar correlation in the Nouna controls (additional file 2). No such relationship was seen between the percentage of CD38^+ ^CD8^+ ^T cells and naïve CD8^+ ^T cells (data not shown).

In Ouagadougou controls, the percentage of naïve CD8^+ ^T cells decreased slightly but significantly with increasing age (additional file 3). This correlation did not reach statistical significance in healthy adults living in the rural setting (Nouna), probably because of the lower number of observations in Nouna compared with Ouagadougou (Table 1). In healthy adults from both settings, the percentage of naïve CD4^+ ^T cells showed a slight but statistically not significant trend to decrease with increasing age (additional file 3). In healthy controls, neither the percentages of activated CD95^+ ^or CD38^+ ^T cells, nor the ABC values of CD95 or CD38, were significantly correlated with age (data not shown). In addition, no significant gender-related differences in the immunologic parameters tested were seen (additional files 4 and 5).

### Differences between HIV-1-infected individuals in rural and urban settings

**Figure 2:**
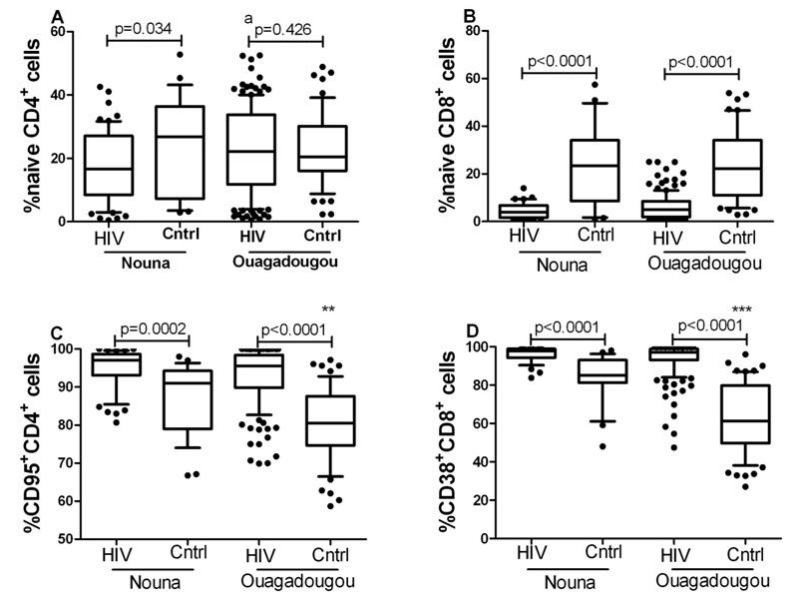
**Percentage of naïve and activated CD4^+ ^and CD8^+ ^T cells in patients and healthy controls**. Whisker plots were used to compare the percentages of naïve and activated CD4^+ ^and CD8^+ ^T cells between HIV-1 infected subjects and healthy controls from the different study sites using the Mann-Whitney U-test. Panels A and B show the comparison of the percentage of naïve CD4^+ ^and CD8^+ ^T cells, respectively, between HIV-1 infected subjects and healthy controls from Nouna (rural) and Ouagadougou (urban). Panels C and D show the comparison of the percentage of CD95^+^CD4^+ ^and of CD38^+^CD8^+ ^T cells, respectively, between HIV-1 infected subjects and healthy controls from Nouna and Ouagadougou. Symbols: (^a^) indicates a statistically significant difference (p < 0.05) between HIV-1 infected subjects in Ouagadougou and HIV-1 infected subjects from Nouna; (** or ***) indicates a statistically significant difference (p < 0.001 and p < 0.0001) between healthy controls in Ouagadougou and healthy controls in Nouna.

As shown in Table 1, patients in the rural setting of Nouna had significantly lower CD4^+ ^T cell counts and slightly higher HIV-1 plasma viral load levels compared with patients in the city of Ouagadougou. Relatively more patients in Nouna were in an advanced disease stage (immunologic class C according to the 1993 classification of the Centers of Disease Control [25]). Consequently, the percentage of circulating naïve T cells was lower in patients living in Nouna compared with those living in Ouagadougou (Figure 2, additional file 1). This difference was statistically significant only for CD4^+ ^T cells and was detectable also in patients with similar CD4^+ ^T cell counts or patients with comparable HIV-1 plasma viral load levels (additional file 6).

When patients with CD4^+ ^T cell counts between 200 and 400 cells/mm^3 ^or patients with HIV-1 plasma viral load levels in the range of 4 to 6 log_10 _RNA copies per ml were compared, a slightly but significantly lower anti-CD95 antibody binding capacity (ABC) of CD95^dim ^CD4^+ ^T cells was observed in patients from Nouna compared with patients from Ouagadogou (additional file 7). In patients with HIV-1 plasma viral load levels in the range of 4 to 6 log_10 _RNA copies per ml, the expression level (ABC) of CD38 on CD38^+ ^CD8^+ ^T cells was slightly higher in the rural than in the urban setting. No significant difference between patients at both study sites was seen regarding ABC of CD95^bright ^CD4^+ ^T cells (additional file 7).

### Differences between HIV-1-infected patients and healthy controls

**Figure 3:**
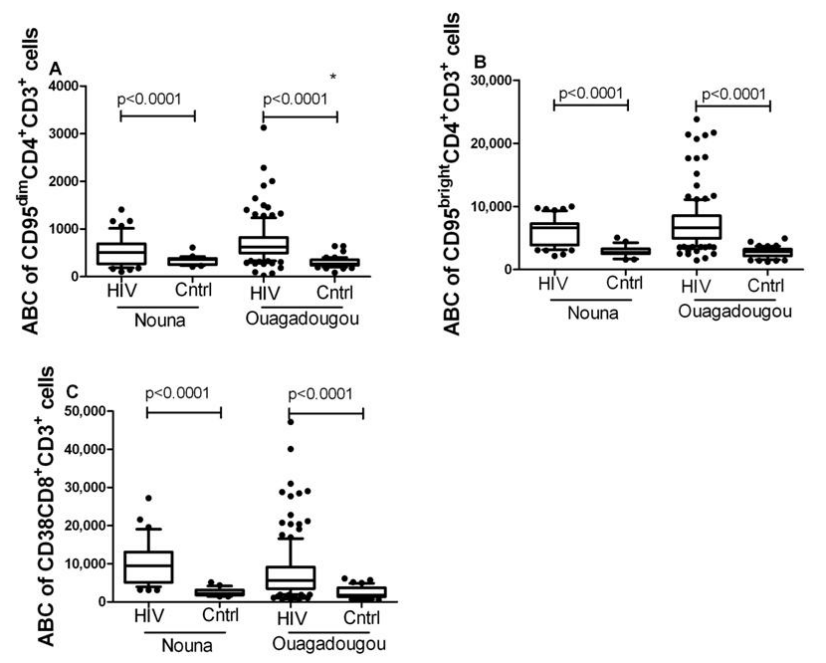
**Expression of T cell activation markers on CD4^+ ^and CD8^+ ^T cells in patients and healthy controls**. Whisker plots were used to compare the antibody-binding capacity (ABC) of CD95^+ ^CD4^+ ^T cells (panel A and B) and CD38^+ ^CD8^+ ^T cells (panel C) between HIV-1 infected subjects and healthy controls from the different study sites using the Mann-Whitney U-test. Panel A shows data on the ABC of CD95^dim ^CD4^+ ^CD3^+ ^cells, panel B on the ABC of CD95^bright^CD4^+ ^CD3^+ ^cells, and panel C shows the comparison of the ABC of CD38 CD8^+ ^CD3^+ ^cells between HIV-1 infected subjects and healthy controls from Nouna (rural) and Ouagadougou (urban). Symbols: (*) indicates statistically significant difference (p < 0.05) between healthy controls in Ouagadougou (urban) and healthy controls in Nouna (rural).

Irrespective of the rural or urban setting the percentage of CD95^+ ^CD4^+ ^and CD38^+ ^CD8^+ ^T cells was significantly higher and the percentage of naïve CD8^+ ^T cells was lower in patients compared to healthy controls (Figure 2, additional file 1). The percentage of naïve CD4^+ ^T cells was significantly lower in HIV-1-infected subjects compared to healthy controls in Nouna but not in Ouagadougou (Figure 2, additional file 1). Anti-CD95 antibody binding capacity (ABC) of CD95^dim ^and CD95^bright ^CD4^+ ^T cells as well as CD38 ABC of CD38^+ ^CD8^+ ^T cells was elevated in patients from both study sites compared to their respective controls (Figure 3, additional file 1).

### Determinants of T cell activation in HIV-1-infected patients

In HIV-1-infected patients living in Ouagadougou, the percentage of CD95^+ ^CD4^+ ^T cells was negatively correlated with CD4^+ ^T cell counts (r = -0.526, R^2 ^= 0.277, p < 0.0001) and positively correlated with HIV-1 plasma viral load levels (r = 0.434, R^2 ^= 0.188, p < 0.0001; additional file 8). In the Nouna patient cohort, a similar relationship between the percentage of CD95^+ ^CD4^+ ^T cells and CD4^+ ^T cell counts was seen (r = -0.512, R^2 ^= 0.263, p = 0.0001) but there was no correlation between CD95^+ ^CD4^+ ^T cells and HIV-1-plasma viral load levels (additional file 8). The percentage of naïve CD45RA^+ ^CCR7^+ ^CD4^+ ^T cells showed a moderate positive correlation with CD4^+ ^T cell counts (statistically significant only in the Ouagadougou cohort) and no clear correlation with HIV-1-plasma viral load levels (additional file 8).

**Figure 4:**
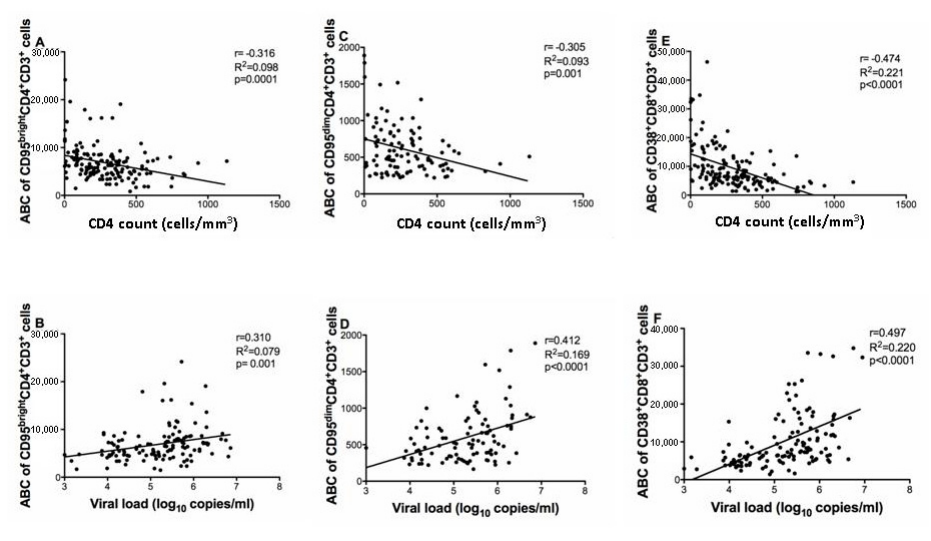
**T cell activation, CD4^+ ^T cell counts, and HIV-1 plasma viral load in the urban setting**. Correlation between activation marker antibody binding capacity (ABC) of peripheral blood T cells and CD4^+ ^T cell counts in 132 treatment-naïve HIV-1 infected patients (panels A, C and E) as well as HIV-1 plasma viral load levels in 125 patients (panels B, D and F) living in Ouagadougou, the capital of Burkina Faso. ABC was derived from the median fluorescence intensity as described in the Methods section. Panel A shows the correlation between the ABC of CD4^+^CD95^bright ^T cells and CD4^+ ^T cell counts, panel B between the ABC of CD4^+^CD95^bright ^T cells and HIV-1 plasma viral load. Panel C depicts the correlation between the ABC of CD4^+^CD95^dim ^T cells and CD4^+ ^T cell counts, and panel D the correlation between the ABC of CD4^+^CD95^dim ^T cells and HIV-1 plasma viral load. Panel E shows the correlation between the ABC of CD8^+^CD38^+ ^T cells and CD4^+ ^T cell counts, and panel F between the ABC of CD8^+^CD38^+ ^T cells and HIV-1 plasma viral load. Correlations between variables were explored by calculation of Pearson's correlation coefficient (r) and the coefficient of determination (R^2^), which are indicated for each analysis.

**Figure 5:**
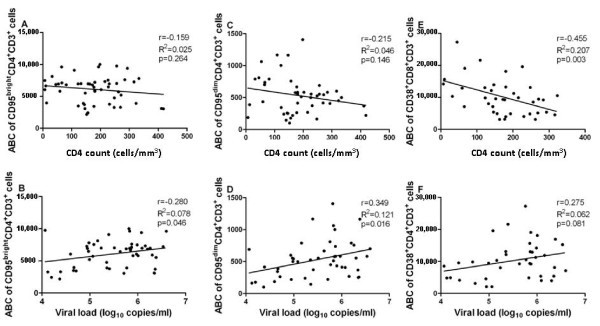
**T cell activation, CD4^+ ^T cell counts, and HIV-1 plasma viral load in the rural setting**. Correlation between activation marker antibody binding capacity (ABC) of peripheral blood T cells and CD4+ T cell counts (panels A, C and E), or HIV-1 plasma viral load levels (panels B, D and F) in 61 treatment-naïve HIV-1 infected patients living in Nouna, rural Burkina Faso. ABC was derived from the median fluorescence intensity as described in the Methods section. Panel A shows the correlation between the ABC of CD4^+^CD95^bright ^T cells and CD4^+ ^T cell counts, and panel B between the ABC of CD4^+^CD95^bright ^T cells and HIV-1 plasma viral load. Panel C depicts the correlation between the ABC of CD4^+^CD95^dim ^T cells and CD4^+ ^T cell counts, and panel D the correlation between the ABC of CD4^+^CD95^dim ^T cells and HIV-1 plasma viral load. Panel E shows the correlation between the ABC of CD8^+^CD38^+ ^T cells and CD4^+ ^T cell counts, and panel F between the ABC of CD8^+^CD38^+ ^T cells and HIV-1 plasma viral load. Correlations between variables were explored by calculation of Pearson's correlation coefficient (r) and the coefficient of determination (R^2^), which are indicated for each analysis.

In patients from Ouagadougou, anti-CD95 antibody binding capacity (ABC) of CD95^bright ^CD4^+ ^T cells was moderately negatively correlated with CD4^+ ^T cell counts (Figure 4A) and positively with HIV-1 plasma viral load levels (Figure 4B). In the Nouna patient cohort, the negative correlation between anti-CD95 ABC of CD95^bright ^CD4^+ ^T cells and CD4^+ ^T cell counts (Figure 5A) was weak and not statistically significant, whereas the relationship with HIV-1 plasma viral load levels was similar to the one seen in patients from Ouagadougou (Figure 5B). Anti-CD95 ABC of CD95^dim ^CD4^+ ^T cells was moderately negatively correlated with CD4^+ ^T cell counts (Figure 4C and 5C), but the level of statistical significance was reached only in patients from Ouagadougou and not from Nouna. In patients from both study sites, anti-CD95 ABC of CD95^dim ^CD4^+ ^T cells showed a somewhat stronger and statistically significant positive correlation with HIV-1 plasma viral load levels (Figure 4D and 5D).

In the CD8^+ ^T cell compartment of the patients from Ouagadougou, the percentage of CD38^+ ^CD8^+ ^T cells was moderately negatively correlated with CD4^+ ^T cell counts (r = -0.365, R^2 ^= 0.133, p < 0.0001) and positively with HIV-1 plasma viral load levels (r = 0.452, R^2 ^= 0.204, p < 0.0001; additional file 8). In the Nouna patient cohort, similar relationships were seen, but did not always reach the level of statistical significance (additional file 8). Anti-CD38 ABC of CD38^+ ^CD8^+ ^T cells showed a significant negative correlation with CD4^+ ^T cell counts in both patient cohorts (Figures 4E and 5E). The positive correlation of anti-CD38 ABC with HIV-1 plasma viral load levels was statistically significant in patients from Ouagadougou (Figure 4F), but not in patients from Nouna (Figure 5F).

## Discussion

Our study reveals that environmental exposure to immune stimulants, which is expected to be more present in rural than in urban settings, influences peripheral blood T cell activation in healthy individuals and in HIV-1-infected patients in west Africa. Healthy adults in Nouna (rural setting) showed a higher percentage of CD95^+ ^CD4^+ ^T cells and an elevated expression level (antibody-binding capacity, ABC) of CD95 on CD95^dim ^CD4^+ ^T cells in peripheral blood compared with healthy adults in Ouagadougou (urban setting). The percentage of CD38^+ ^CD8^+ ^T cells was also higher and the expression of CD38 on CD38^+ ^CD8^+ ^T cells was increased. Thus, healthy adults living in the rural setting harbour more peripheral blood T cells showing signs of cellular activation compared with individuals living in the urban setting.

Both the expression of activation markers and the distribution pattern of T cell maturation phenotypes may be a surrogate parameter for environmentally driven immuno-senescence [11,26,27]. A previous study from Ethiopia has shown comparable CD4^+ ^T cell counts but higher CD8^+ ^T cell counts in healthy male subjects living in Akaki (urban) and Wonji (rural). In this setting, both the proportions of activated CD38^+ ^peripheral blood T cells and of naïve CD45RA^+ ^CD27^+ ^T cells were similar [27].

In our own study, differences in T cell activation between the rural and the urban populations were not accompanied by significant differences in patterns of peripheral T cell maturation. The percentages of naïve CD4^+ ^and CD8^+ ^T cells were not significantly higher in healthy adults living in Ouagadougou than in those living in Nouna. The percentage of activated CD95^+ ^CD4^+ ^T cells was negatively correlated with the percentage of naïve CD4^+ ^T cells (additional file 2). No such correlation was seen between naïve and activated CD8^+ ^T cells (data not shown). Yet the linear decrease in the proportion of naïve T cells with increasing age as described for Europeans [11] seems to be present also in the west African population studied by us (additional file 3). Neither the expression of CD95^+ ^on CD4^+ ^T cells, nor of CD38^+ ^on CD8^+ ^T cells, was significantly correlated with age (data not shown). In contrast to published observations from Dakar, Senegal [28], we did not see a significant gender-related difference in T cell activation or maturation in our study populations (additional files 4 and 5).

In our study, the observed increase in CD95^+ ^T cells in healthy adults living in Nouna compared with individuals living in Ouagadougou was not caused by a reduction in the proportion of CD95^dim ^naïve CD4^+ ^T cells, but involved changes in CD95 receptor density on the naïve cell population. T cell surface markers like CD95 on CD4^+ ^and CD38 on CD8^+ ^T cells seem to be more sensitive than the T cell maturation phenotypes towards the mechanisms that mediate environmental influences on the human peripheral blood T cell pool. We had observed high prevalences of herpes viruses, dengue and hepatitis B virus in the study area [29], and had seen specific CD4^+ ^T cell responses against Plasmodium falciparum in 14 out of 17 healthy adults in Nouna (data not shown). T cell responses to viral infection would be expected to increase predominantly the expression of CD38 on CD8^+ ^T cells [30]. In contrast, CD4^+ ^T cell responses to Plasmodium falciparum may increase the expression of CD95 on resting naïve cells by unspecific "bystander" activation due to an increased production of interferon gamma [31].

One could thus hypothesize that circulating naïve T cells in healthy adults living in the rural setting may become constitutively CD95^+ ^in response to environmental stimuli. It is not known whether increased CD95 expression on these cells is also increasing the sensitivity for CD95-induced apoptosis or altering other functional T cell properties. The CD95 signalling cascade has pleiotropic effects on peripheral blood T cells. While high signal intensities may silence T cell receptor-induced activation (thus inducing T cell anergy) or may cause activation-induced cell death via apoptosis, low signal intensities especially in resting T cells may exert co-stimulatory effects [32]. An increase in the expression level of CD95 on CD4^+ ^lymphocytes may therefore lower the general activation threshold of resting naïve CD4^+ ^T cells.

Are environmental factors acting in a similar way in HIV-1-infected adults? There was no relevant and statistically significant difference in the level of T cell activation and T cell maturation between patients from both study sites. Relatively more patients living in the rural setting of Nouna were in an advanced disease stage. The small but significant difference in the percentage of CD45RA^+ ^CCR7^+ ^naïve CD4^+ ^lymphocytes between patients living in the two settings is best explained by this fact. When patients with HIV-1 plasma viral load levels of 4 to 6 log_10 _RNA copies per ml at both study sites were compared, the expression level of CD38 on CD8^+ ^T cells was slightly higher in the rural than in the urban setting. In the CD8^+ ^T cell compartment, environmental influences may therefore add to HIV-induced changes of the activation status.

Patients with comparable CD4^+ ^T cell counts (200 to 400 cells/mm^3^) or HIV-1 plasma viral load levels (4 to 6 log_10 _RNA copies per ml) in the rural setting showed lower percentages of naïve CD4^+ ^T cells than those in the urban setting with similar disease activity. These observations indicate that environmental determinants of T cell activation may act also in the CD4^+ ^T cell compartment of HIV-1-infected patients living in the rural setting.

Anti-CD95 ABC of CD95^dim ^CD4^+ ^T cells were slightly lower in the Nouna patient cohort than in patients from Ouagadougou. It is tempting to speculate that, due to environmental trigger mechanisms, CD95^dim ^naïve CD4^+ ^T cells in HIV-1-infected individuals living in the rural setting are more prone to get activated or to undergo programmed cell death (apoptosis) upon low level CD95 triggering than in patients who live in an urban environment. These cells may then either be lost due to an increased susceptibility for CD95-induced apoptosis, which is a known feature of peripheral blood T cells in HIV-1-infected patients [15], or due to accelerated maturation.

We were not able to incorporate simultaneous T cell phenotyping and assessment of T cell function into our study protocol. However, elevated immune activation, together with increased expression of CD95 and increased spontaneous T cell apoptosis, has been described in a cohort of healthy women from Nairobi, Kenya [26], as well as in healthy adults from Ghana [33]. Such investigations should be included in future studies in order to clarify the functional capacity of CD95^dim ^naïve CD4^+ ^T cells both in HIV-1-infected individuals and in healthy controls living under different environmental conditions.

Infection with HIV-1, however, exerts a much stronger effect on T cell activation than any differences in environmental conditions. Our results confirm the well-known finding that peripheral blood T cells of HIV-1-infected patients show a higher expression of activation markers compared with healthy controls of similar age [3-5]. The percentage of CD95^+ ^CD4^+ ^and CD38^+ ^CD8^+ ^T cells, anti-CD95 ABC of CD95^dim ^and CD95^bright ^CD4^+ ^T cells and CD38 ABC of CD38^+ ^CD8^+ ^T cells were all elevated in patients compared with controls at both study sites. This observation was not accompanied by similar changes in the expression of T cell maturation markers. Only in Nouna the percentage of naïve CD4^+ ^T cells was significantly lower in HIV-1-infected subjects than in healthy controls. A possible explanation for this observation may be the aforementioned (hypothetical) CD95-mediated loss of CD95^dim ^naïve CD4^+ ^T cells in HIV-1-infected individuals living in the rural setting. The difference in age between patients and controls does not explain the observed differences in the percentage of naïve T cells.

Absolute numbers of naïve CD4^+ ^T cells in HIV-1-infected subjects (mean±standard deviation: 32.5 ± 33.6 cells/mm^3 ^in Nouna, 72.3 ± 79.3 cells/mm^3 ^in Ouagadougou) were significantly lower than the population-specific reference range in our study area (5^th ^to 95^th ^percentile: 35 to 496 cells/mm^3^) [10]. In the CD8^+ ^T cell compartment, the proportion of naïve lymphocytes was significantly reduced in HIV-1-infected patients compared with healthy controls at both study sites. The absolute counts of naïve CD8^+ ^T cells (mean±standard deviation: 43.0 ± 49.4 cells/mm^3 ^in Nouna; 53.0 ± 61.6 cells/mm^3 ^in Ouagadougou) were significantly lower than those described for healthy adults in our study area (5^th ^to 95^th ^percentile: 36 to 363 cells/mm^3^) [10].

Chronic immune activation observed in HIV-1-infected subjects has been shown to be associated both with low CD4^+ ^T cell counts and high HIV-1 RNA levels [23,34]. CD38, the most extensively studied activation marker in HIV-1-infected subjects, had been reported to be upregulated on CD8^+ ^T cells in association with disease progression [12-14,35-38]. The degree of correlation between CD38 expression on CD8^+ ^T cells and HIV-1 plasma viral load in our study was moderate but comparable to other African studies [12,14].

Upregulation of the T cell activation marker CD95 on CD4^+ ^had previously been reported on CD4^+ ^T cells of HIV-1 infected subjects in industrialized countries and was also associated with disease progression [15,17,21,39]. The small but significant increase of CD95 ABC of both CD95^dim ^and CD95^bright ^CD4^+ ^T cells with increasing viral load indicates that CD95 expression in HIV-1 infected patients may be partially driven by viral replication. Circulating CD95^+ ^CD4^+ ^T cells in patients with advanced disease are mainly CD95^bright ^memory cells, and disappearance of CD95^dim ^T cells in these patients usually parallels the depletion of CD45RA^bright ^CCR7^+ ^naïve T lymphocytes [21].

In HIV-1-infected subjects living in Burkina Faso, T cell activation levels increased and proportions of naïve T cells decreased both with increasing HIV-1 plasma viral load and decreasing CD4^+ ^T cell counts. A significant correlation (r = -0.456, R^2 ^= 0.19, p < 0.0001; data not shown) was also observed between absolute CD4^+ ^T cell counts and HIV-1 plasma viral load levels in Ouagadougou. However, no causal relationship can be inferred from our data between these parameters and changes in T cell activation and maturation patterns in HIV-1-infected patients. Skewed maturation and increased activation of peripheral blood T cells in HIV-1-infected patients compared with healthy controls may be explained by HIV-induced T cell loss. This may lead to peripheral homeostatic expansion causing an increase in the expression levels of CD95 or CD38 on circulating naïve T cells as a sign of cellular cycling [40]. Differences in the exposure to other viral infections than HIV is an unlikely cause of the deviations observed in the CD4^+ ^and CD8^+ ^T cell compartments in these individuals. HIV replication acts as a continuous T cell stimulus, but is expected to increase primarily the expression of CD38 on CD8^+ ^T cells [35].

These observations point to a common problem in flow cytometric analysis of T cell activation markers in HIV-infected individuals. In the individual patient with a high CD4^+ ^T cell count, an elevated expression of CD95 on CD95^dim ^CD4^+ ^T cells and of CD38 on CD38^+ ^CD8^+ ^T cells most probably indicates a high HIV-1 plasma viral load. In the lymphopenic patient with advanced disease and a low CD4^+ ^T cell count, phenotypic effects of cell cycling due to homeostatic autoproliferation may influence the expression levels of these molecular markers of T cell activation.

## Conclusions

We conclude from our data that environmentally triggered immune activation contributes to the increased expression of the T cell activation markers, CD95 and CD38, on peripheral blood T cells from healthy adults living in rural versus urban settings in Burkina Faso. Environmentally induced T cell activation is also detectable in HIV-1-infected individuals, albeit at a much lower level than the changes caused by high HIV-1 plasma viral load and/or HIV-induced T cell loss. The observed variations in T cell activation levels and the proportion of naïve T cells in our study patients, however, are not explained by differences in CD4^+ ^T cell counts and HIV-1 plasma viral load levels alone. At least under the given environmental conditions in west Africa, where people are continuously exposed to T cell stimulating agents, such as viral antigens or parasite infestations [29,41-46], the clinical value of additional measurements of these immune markers in HIV-1-infected patients remains to be demonstrated.

## Competing interests

The authors declare that they have no competing interests.

## Authors' contributions

FT organized recruitment of patients and healthy controls in Nouna and Ouagadougou, collected clinical and laboratory data, performed immunological and virological measurements in the laboratories of the CRSN and CHUYO with the help of LS and BC, and wrote the first draft of the manuscript. TB, FN and HGK designed the study, evaluated interim findings, and assisted in revising subsequent versions of the manuscript. All authors have read and approved the final version of this manuscript.

## Supplementary Material

Additional file 1 **Supplementary material a (MS Word)**. Comparison of clinical and immunological parameters between HIV-1-infected and healthy adults living in rural and urban Burkina Faso. Click here for file

Additional file 2 **Supplementary material b (MS PowerPoint)**. Correlation between the percentage of activated T cells and the percentage of naïve T cells in healthy adults in Nouna and Ouagadougou. Click here for file

Additional file 3 **Supplementary material c (MS PowerPoint)**. Correlation between the percentage of naïve T cells and age in healthy adults in Nouna and Ouagadougou. Click here for file

Additional file 4 **Supplementary material d (MS PowerPoint)**. Gender-related differences in the percentage of naïve and activated T cells or expression levels of T cell activation markers in healthy adults living in Nouna. Click here for file

Additional file 5 **Supplementary material e (MS PowerPoint)**. Gender-related differences in the percentage of naïve and activated T cells or expression levels of T cell activation markers in healthy adults living in Ouagadougou. Click here for file

Additional file 6 **Supplementary material f (MS PowerPoint)**. Differences in the percentage of naïve and activated T cells between subgroups of HIV-1-infected patients living in Nouna and Ouagadougou. Click here for file

Additional file 7 **Supplementary material g (MS PowerPoint)**. Differences in the expression level of T cell activation markers between subgroups of HIV-1-infected patients living in Nouna and Ouagadougou. Click here for file

Additional file 8 **Supplementary material h (MS Word)**. Correlation analysis between clinical and immunological parameters in HIV-1-infected adults living in Nouna and Ouagadougou. Click here for file
