# Supplementary material for: Activation and maturation of peripheral blood T cells in HIV-1-infected and HIV-1-uninfected adults in Burkina Faso: a cross-sectional study
Source: J Int AIDS Soc. 2011 Dec 17;14:57. doi: 10.1186/1758-2652-14-57 (PMC3281784; doi:10.1186/1758-2652-14-57)
Supplement: Additional file 1 — Supplementary material a (MS Word). Comparison of clinical and immunological parameters between HIV-1-infected and healthy adults living in rural and urban Burkina Faso. [file 1758-2652-14-57-S1.DOC]

**Supplementary material a.** Comparison of clinical and immunological parameters between HIV-1-infected and healthy adults living in rural and urban Burkina Faso

All patients were “treatment-naïve”, i.e., without known or reported previous exposure to single or combined antiretroviral drugs or highly active antiretroviral combination therapy (HAART). If not otherwise indicated, median values are shown with 10^th^ and 90^th^ percentiles in parentheses. Selected tests were not performed in all patients or controls; the actual numbers of observations are indicated in brackets. The Mann-Whitney U-test was used to compare patients and controls in the rural and urban setting, as well as patients in the rural and urban setting and controls in the rural and the urban setting separately. Abbreviations: ABC – antibody binding capacity expressed as median binding sites per cell; CDC – Centers of Disease Control and Prevention immunologic staging; n.d. – not done; p – p-value for the difference between patients and controls.

|  | Nouna (rural) | | *p* | Ouagadougou (urban) | | *p* |
| --- | --- | --- | --- | --- | --- | --- |
|  | Patients | Controls |  | Patients | Controls |  |
| n | 61 | 26 | - | 137 | 63 | - |
| Female [n = ; (%)] | 44 (72%) | 16 (61%) | <0.0001 | 98 (72%) | 25 (40%)* | <0.0001 |
| Age [years; median, range] | 35 (30-43) | 27 (19-35) | <0.0001 | 35 (23-48) | 28 (20-35) | <0.0001 |
| CD4^+^ T cell count [µl^-1^] | 174 (33-314) | n.d. | - | 256 (118-387)b | n.d. | - |
| CDC-stage A [n = ; (%)] | 01 (02%) | n.d. | - | 22 (16%)c | n.d. | - |
| CDC-stage B [n = ; (%)] | 23 (38%) | n.d. | - | 55 (40%)c | n.d. | - |
| CDC-stage C [n = ; (%)] | 37 (60%) | n.d. | - | 60 (44%)c | n.d. | - |
| HIV-1 plasma viral load (log_10_ copies ml^-1^] | 5.8 (4.6-6.6) | n.d. | - | 5.4 (4.0-6.2)b  [n=125] | n.d. | - |
| Naive CD45RA^+^ CCR7^+^ CD4^+^ T cells  [% of CD4^+^ lymphocytes] | 16 (3-36)  [n=50] | 26 (4-43) | 0.03 | 22 (4-40)a | 23 (11-38)  [n=62] | 0.4 |
| activated CD4^+^ CD95^+^ [% of CD3^+^CD4^+^ T cells] | 97 (91-100)  [n=53] | 91 (77-98) | 0.0002 | 96 (83-100) | 82 (68-95)**  [n=61] | <0.0001 |
| CD95 ABC of CD3^+^CD4^+^CD95^dim^ T cells | 555 (267-1168) | 326 (193-432) | <0.0001 | 669 (378-1275) | 287 (193-432)* | <0.0001 |
| CD95 ABC of CD3^+^CD4^+^CD95^bright^ T cells | 7201 (3763-9363) | 3655 (2598-5142) | <0.0001 | 6383 (3639-10507) | 3075 (2013-4511) | <0.0001 |
| Naive CD45RA^+^ CCR7^+^CD8^bright^ T cells  [% of CD8^bright^ lymphocytes] | 4 (1-9) | 27 (6-37) | <0.0001 | 7 (1-16) | 21 (5-44)  [n=62] | <0.0001 |
| activated CD8^+^ CD38^+^ (% of CD3^+^CD8^+^ T cells) | 98 (90-100)  [n=53] | 83 (63-98) | <0.0001 | 97 (84-100) | 63 (36-83)*** | <0.0001 |
| CD38 ABC of CD3^+^CD8^+^CD38^+^ T cells | 9685 (3081-20327) | 2098 (1266-4783) | <0.0001 | 7401 (3625-19860) | 548 (195-1753)*** | <0.0001 |

ap <0.05, bp <0.001, cp <0.001 indicating statistically significant differences between HIV-1 infected subjects in Ouagadougou compared with HIV-1 infected subjects from Nouna

*p <0.05; **p <0.01; ***p <0.001, indicating statistically significant differences between healthy controls in Ouagadougou compared with healthy controls in Nouna
