# Supplementary material for: Activation and maturation of peripheral blood T cells in HIV-1-infected and HIV-1-uninfected adults in Burkina Faso: a cross-sectional study
Source: J Int AIDS Soc. 2011 Dec 17;14:57. doi: 10.1186/1758-2652-14-57 (PMC3281784; doi:10.1186/1758-2652-14-57)
Supplement: Additional file 2 — Supplementary material b (MS PowerPoint). Correlation between the percentage of activated T cells and the percentage of naïve T cells in healthy adults in Nouna and Ouagadougou. [file 1758-2652-14-57-S2.PPT]

## Slide 1
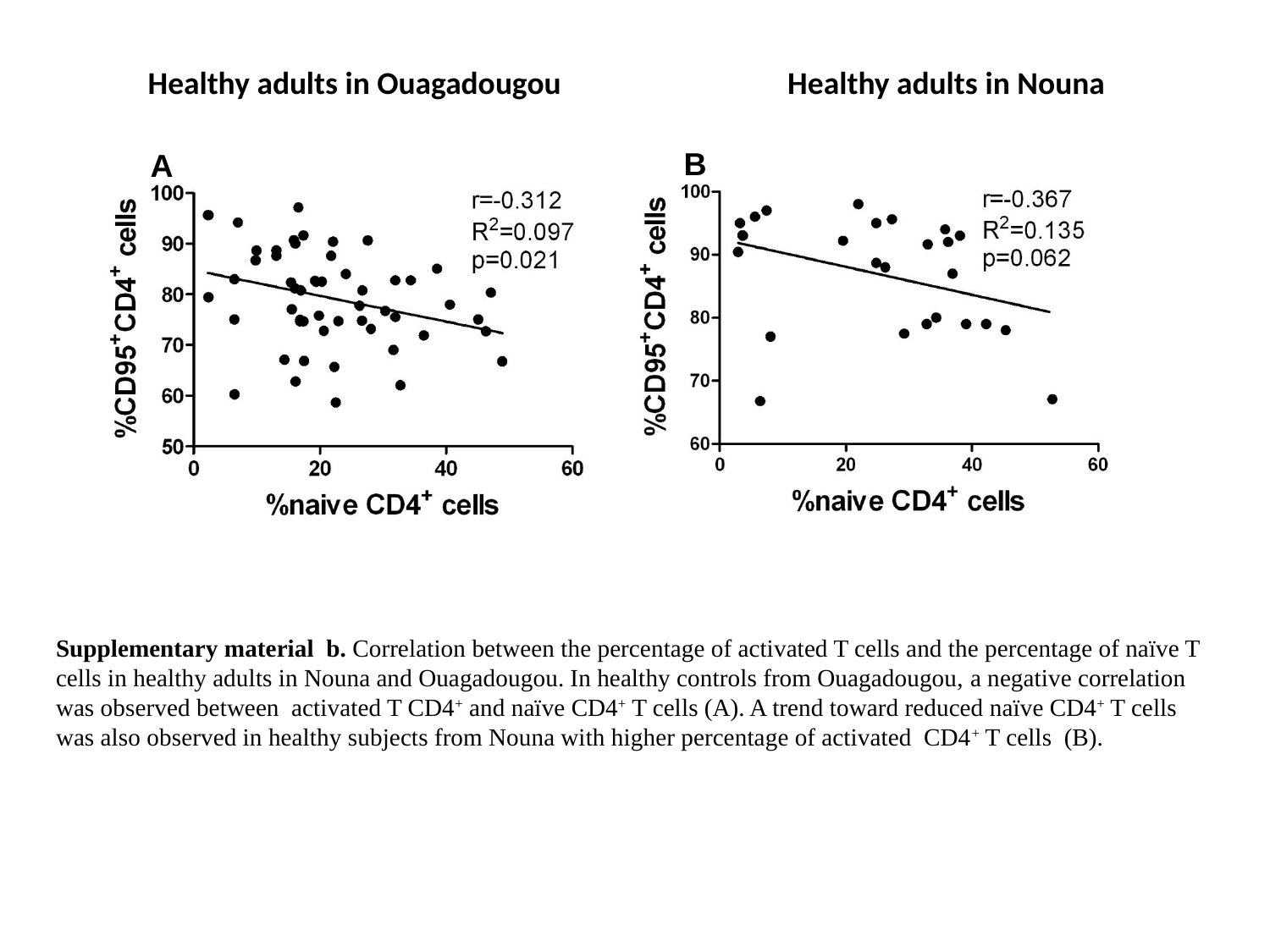

Healthy adults in Ouagadougou
Healthy adults in Nouna
B
A
Supplementary material b. Correlation between the percentage of activated T cells and the percentage of naïve T cells in healthy adults in Nouna and Ouagadougou. In healthy controls from Ouagadougou, a negative correlation was observed between activated T CD4+ and naïve CD4+ T cells (A). A trend toward reduced naïve CD4+ T cells was also observed in healthy subjects from Nouna with higher percentage of activated CD4+ T cells (B).
