# Supplementary material for: Activation and maturation of peripheral blood T cells in HIV-1-infected and HIV-1-uninfected adults in Burkina Faso: a cross-sectional study
Source: J Int AIDS Soc. 2011 Dec 17;14:57. doi: 10.1186/1758-2652-14-57 (PMC3281784; doi:10.1186/1758-2652-14-57)
Supplement: Additional file 3 — Supplementary material c (MS PowerPoint). Correlation between the percentage of naïve T cells and age in healthy adults in Nouna and Ouagadougou. [file 1758-2652-14-57-S3.PPT]

## Slide 1
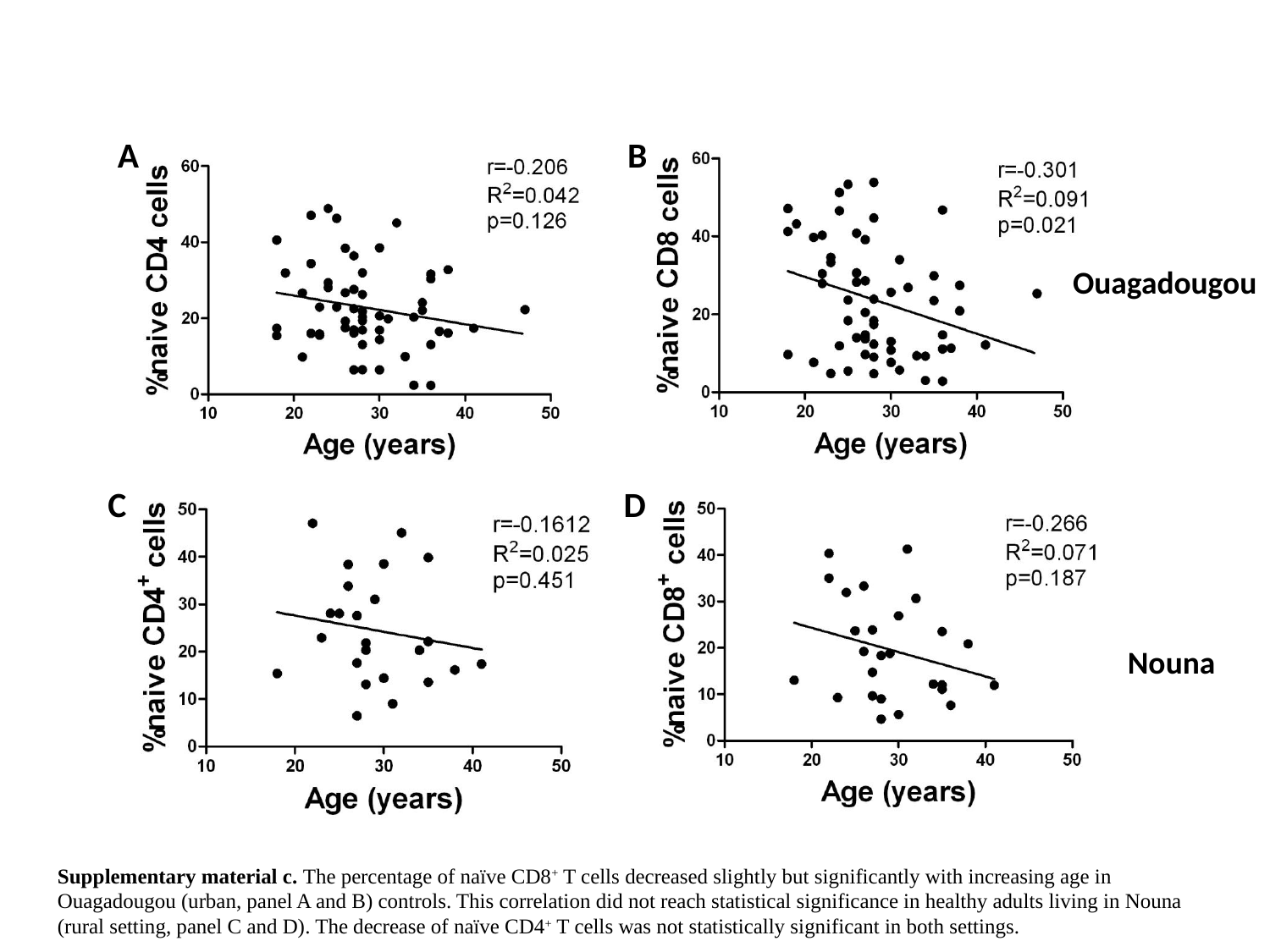

A
B
Ouagadougou
C
D
Nouna
Supplementary material c. The percentage of naïve CD8+ T cells decreased slightly but significantly with increasing age in Ouagadougou (urban, panel A and B) controls. This correlation did not reach statistical significance in healthy adults living in Nouna (rural setting, panel C and D). The decrease of naïve CD4+ T cells was not statistically significant in both settings.
