# Supplementary material for: Activation and maturation of peripheral blood T cells in HIV-1-infected and HIV-1-uninfected adults in Burkina Faso: a cross-sectional study
Source: J Int AIDS Soc. 2011 Dec 17;14:57. doi: 10.1186/1758-2652-14-57 (PMC3281784; doi:10.1186/1758-2652-14-57)
Supplement: Additional file 4 — Supplementary material d (MS PowerPoint). Gender-related differences in the percentage of naïve and activated T cells or expression levels of T cell activation markers in healthy adults living in Nouna. [file 1758-2652-14-57-S4.PPT]

## Slide 1
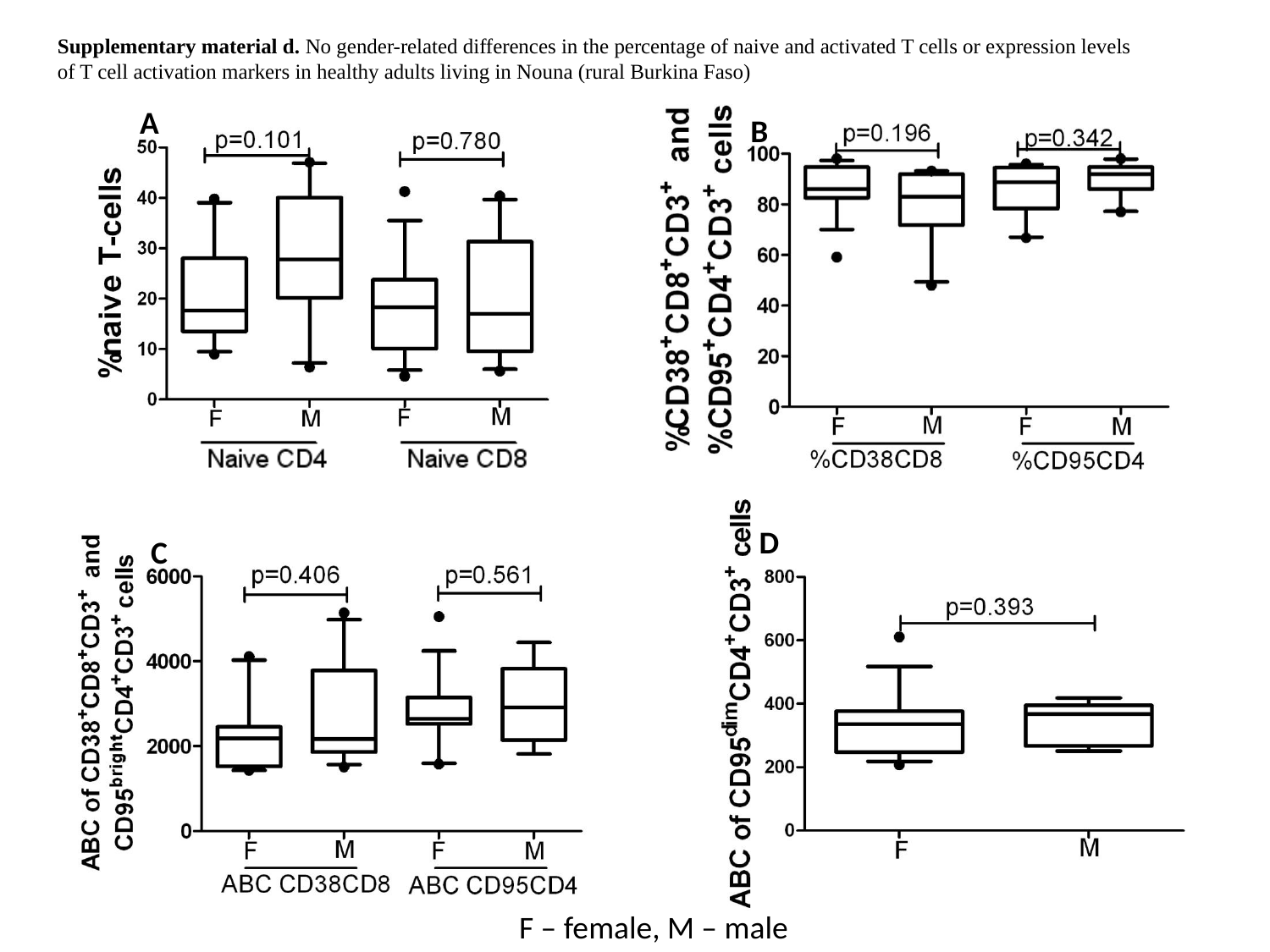

Supplementary material d. No gender-related differences in the percentage of naive and activated T cells or expression levels of T cell activation markers in healthy adults living in Nouna (rural Burkina Faso)
B
A
D
C
F – female, M – male
