# Supplementary material for: Activation and maturation of peripheral blood T cells in HIV-1-infected and HIV-1-uninfected adults in Burkina Faso: a cross-sectional study
Source: J Int AIDS Soc. 2011 Dec 17;14:57. doi: 10.1186/1758-2652-14-57 (PMC3281784; doi:10.1186/1758-2652-14-57)
Supplement: Additional file 5 — Supplementary material e (MS PowerPoint). Gender-related differences in the percentage of naïve and activated T cells or expression levels of T cell activation markers in healthy adults living in Ouagadougou. [file 1758-2652-14-57-S5.PPT]

## Slide 1
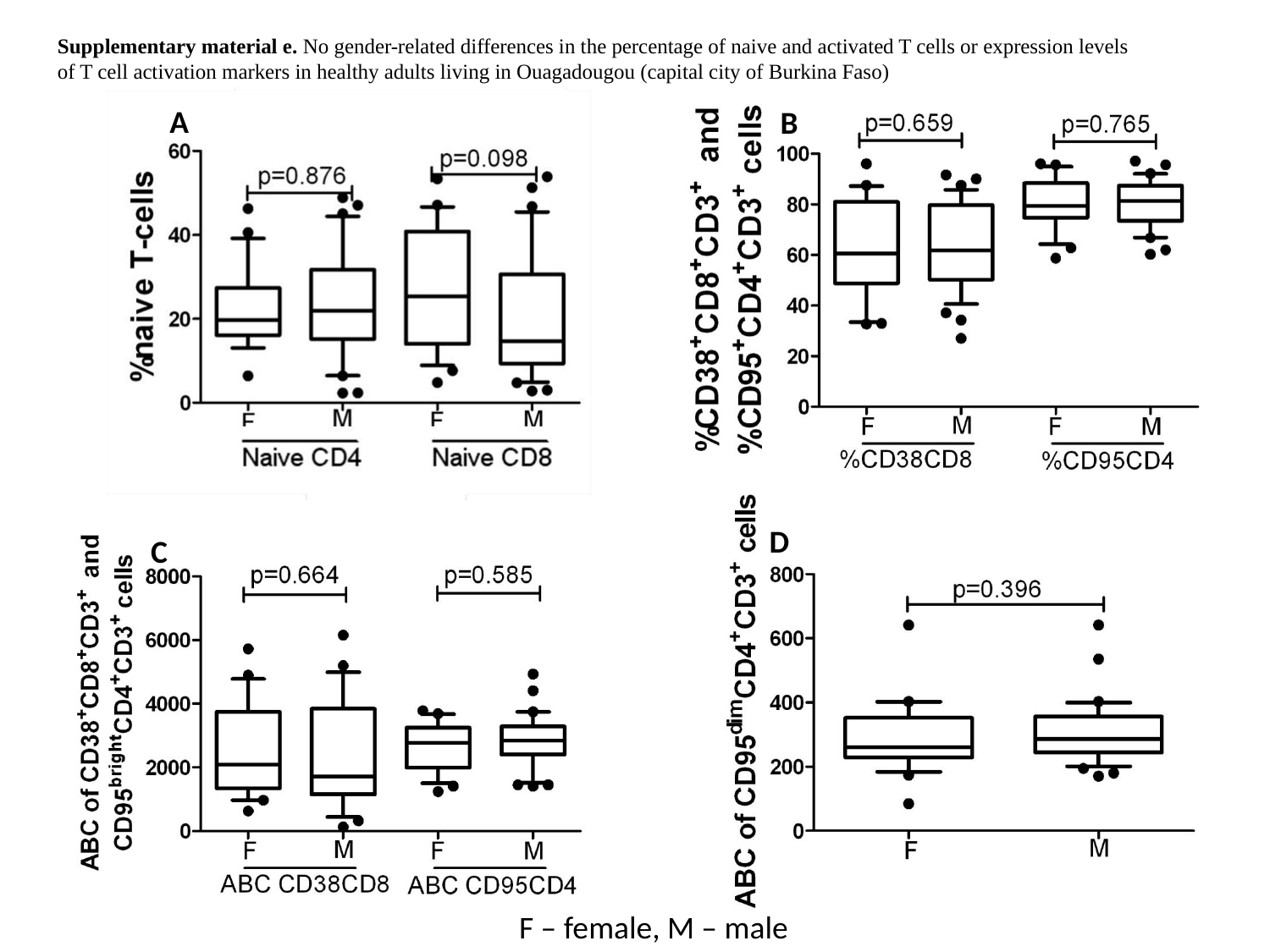

Supplementary material e. No gender-related differences in the percentage of naive and activated T cells or expression levels of T cell activation markers in healthy adults living in Ouagadougou (capital city of Burkina Faso)
A
B
D
C
F – female, M – male
