# Supplementary material for: Activation and maturation of peripheral blood T cells in HIV-1-infected and HIV-1-uninfected adults in Burkina Faso: a cross-sectional study
Source: J Int AIDS Soc. 2011 Dec 17;14:57. doi: 10.1186/1758-2652-14-57 (PMC3281784; doi:10.1186/1758-2652-14-57)
Supplement: Additional file 6 — Supplementary material f (MS PowerPoint). Differences in the percentage of naïve and activated T cells between subgroups of HIV-1-infected patients living in Nouna and Ouagadougou. [file 1758-2652-14-57-S6.PPT]

## Slide 1
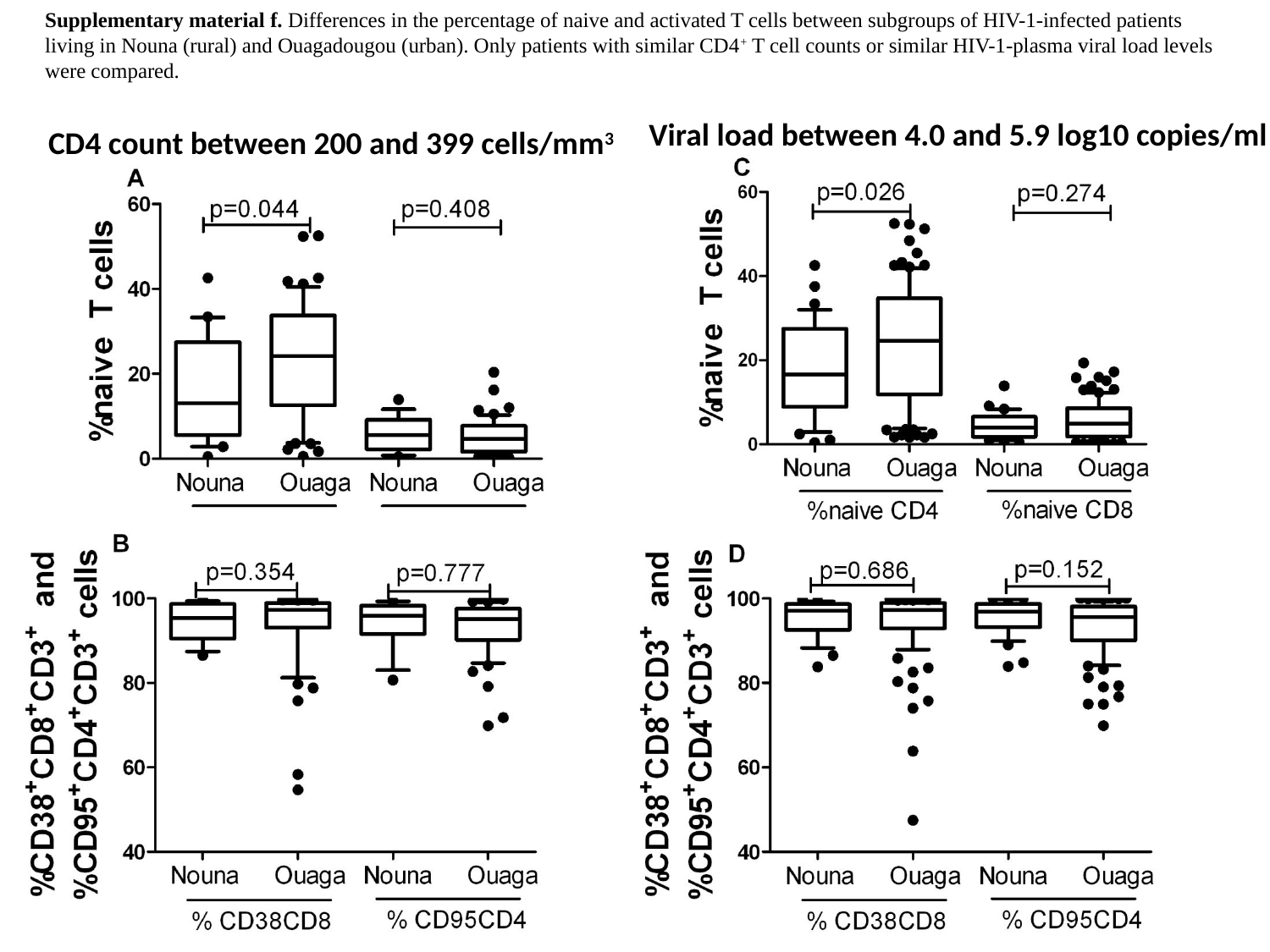

Supplementary material f. Differences in the percentage of naive and activated T cells between subgroups of HIV-1-infected patients living in Nouna (rural) and Ouagadougou (urban). Only patients with similar CD4+ T cell counts or similar HIV-1-plasma viral load levels were compared.
Viral load between 4.0 and 5.9 log10 copies/ml
CD4 count between 200 and 399 cells/mm3
