# Supplementary material for: Activation and maturation of peripheral blood T cells in HIV-1-infected and HIV-1-uninfected adults in Burkina Faso: a cross-sectional study
Source: J Int AIDS Soc. 2011 Dec 17;14:57. doi: 10.1186/1758-2652-14-57 (PMC3281784; doi:10.1186/1758-2652-14-57)
Supplement: Additional file 8 — Supplementary material h (MS Word). Correlation analysis between clinical and immunological parameters in HIV-1-infected adults living in Nouna and Ouagadougou. [file 1758-2652-14-57-S8.DOC]

**Supplementary material h.** Results of correlation analysis between clinical and immunological parameters in HIV-1-infected adults living in rural and urban Burkina Faso

Correlations between the frequencies of naïve or activated T cells and CD4^+^ T cell counts or HIV-1 plasma viral load were explored separately in the patient cohorts from Nouna and Ouagadougou by linear regression analysis using Pearson’s correlation coefficients (r) and the coefficients of determination (R^2^).

Abbreviations: p – p value for the association between selected parameters

|  | Nouna (rural) | | Ouagadougou (urban) | |
| --- | --- | --- | --- | --- |
| Parameters | CD4 cell count | Viral load | CD4 cell count | Viral load |
| Naïve CD45RA^+^ CCR7^+^ CD4^+^ T-cells  [% of CD4^+^ lymphocytes] | r=0.274; R^2^=0.075; p=0.053 | r=-0.110; R^2^=0.000; p=0.962 | r=0.238; R^2^=0.056; p=0.005 | r=-0.166; R^2^=0.027; p=0.051 |
| activated CD4^+^ CD95^+^ [% of CD3^+^CD4^+^ T-cells] | r=0.512; R^2^=0.263; p=0.0001 | r=-0.005; R^2^=0.012; p=0.446 | r=-0.526; R^2^=0.277; p<0.0001 | r=0.434; R^2^=0.188; p<0.0001 |
| Naïve CD45RA^+^ CCR7^+^CD8^bright^ T-cells  [% of CD8^bright^ lymphocytes] | r=0.263; R^2^=0.069; p=0.064 | r=-0.117; R^2^=0.013; p=0.416 | r=0.432; R^2^=0.186; p<0.0001 | r=-0.300; R^2^=0.090; p=0.0006 |
| activated CD8^+^ CD38^+^ (% of CD3^+^CD8^+^ T-cells) | r=-0.212; R^2^=0.082; p=0.079 | r=0.371; R^2^=0.138; p=0.019 | r=-0.365; R^2^=0.133; p<0.0001 | r=0.452; R^2^=0.204; p<0.0001 |
